# An Angle- and Polarization-Selective Dual-Wavelength Narrowband Thermal Emitter for Infrared Multilevel Encryption

**DOI:** 10.34133/research.0719

**Published:** 2025-06-02

**Authors:** Xuan Zhang, Zhengji Wen, Qingzi Li, Zhanpeng Wang, Yusong Sheng, Zhengai Chen, Wenchao Zhao, Meng Guo, He Zhu, Ning Dai, Yuchuan Shao

**Affiliations:** ^1^ Hangzhou Institute for Advanced Study, University of Chinese Academy of Sciences, Hangzhou 310024, China.; ^2^ Laboratory of Thin Film Optics, Key Laboratory of Materials for High Power Laser, Shanghai Institute of Optics and Fine Mechanics, Chinese Academy of Sciences, Shanghai 201800, China.; ^3^ University of Chinese Academy of Sciences, Beijing 100049, China.; ^4^ State Key Laboratory of Infrared Physics, Shanghai Institute of Technical Physics, Chinese Academy of Sciences, Shanghai 200083, China.; ^5^ School of Electronic Information, Huzhou College, Huzhou 313000, China.; ^6^ Jiangsu Collaborative Innovation Center of Photovoltaic Science and Engineering, Changzhou 213164, China.

## Abstract

The explosive growth of data has intensified challenges to information security, spurring a critical need for advanced encryption technologies, and relying solely on digital encryption still leaves information vulnerable to interception and leakage during transmission. Therefore, encryption technologies that combine digital algorithms with physical keys to further enhance information security are widely studied. In this work, we present an angle- and polarization-selective dual-wavelength long-wavelength infrared narrowband thermal emitter for infrared encryption–decryption applications. The thermal emitter is composed of an epsilon-near-zero material upon a metallic layer, designed to enable the excitation of the Berreman mode and asymmetric Fabry–Pérot resonance simultaneously. Numerical simulations combined with the transfer matrix method are employed to analytically investigate the optical responses, demonstrating good agreement with experimental results. Moreover, a robust multilevel cryptographic communication system is developed, utilizing the thermal emitter’s imaging results as the physical-layer key to enable highly efficient information encryption and decryption. We anticipate that the proposed thermal emitters will pave the way for realizing relevant applications in various information encryption devices.

## Introduction

The explosive growth of data has exacerbated information security challenges, driving widespread attention to encryption technology as a critical component for information security [1,2]. To enhance the security of data, researchers have explored various digital cryptographic techniques [3–5], including the Data Encryption Standard as the first widely adopted symmetric algorithm, the Rivest–Shamir–Adleman algorithm for asymmetric encryption and digital signatures, and the Advanced Encryption Standard as the modern standard for symmetric encryption. However, relying solely on digital encryption methods is insufficient to completely prevent information interception and leakage during transmission. This limitation has led to the emergence of joint encryption techniques that combine both the digital algorithms and physical keys to further enhance security. The optical key is one of the important physical keys, serving as a pivotal component in advanced encryption systems [6]. Previous studies have shown that certain smart luminescent compounds exhibit phosphorescent changes under various conditions, such as thermochromic, photochromic, solvatochromic, mechanochromic, and electrochromic phosphorescence [7–13]. Additionally, metasurface technology has been applied for information encryption by modulating the wavelength [14–16], amplitude [17–19], polarization [20–23], and orbital angular momentum of light [24,25]. Multifunctional deep-subwavelength thin films [26–29], integrated with fluorescence, circular polarization, full-color emission, and time-resolved characteristics, have also substantially broadened the scope of encryption technologies.

While extensive research has been conducted in the visible light spectrum [30–32], new areas of optical information encryption remain a challenge, particularly in the infrared (IR) spectrum. Thermal-radiation-based encryption in the IR range has emerged as an effective alternative, leveraging the advantage of IR data visualization [33]. With advancements in thermal sensing equipment, temperature-responsive IR thermal radiation can be detected using IR cameras, allowing for the direct storage of digital information. Information encoding is realized by modulating thermal radiation intensity through precise control of radiation polarization. Moreover, the integration of multichannel independent optical recording techniques can substantially improve both encryption robustness and storage capacity. Whereas certain smart luminescent compounds demonstrate tunable phosphorescent properties, their implementation in optical encryption systems faces several critical limitations, including environmental instability, restricted operational spectral bandwidth, and susceptibility to photodegradation under continuous light exposure [34–36]. Similarly, although thermal emitters fabricated through costly high-precision metasurface techniques provide exceptional control over thermal radiation, they face substantial challenges in terms of scalable production and cost efficiency [37–41].

In this work, we present a cost-effective, lithography-free, wafer-scale thermal emitter with angle- and polarization-selective dual-wavelength narrowband characteristics enabling IR information encryption and decryption. This emitter is composed of an epsilon-near-zero (ENZ) material upon a metallic layer, and it exhibits angle and polarization selectivity, which are based on absorption enhancement near the longitudinal optical (LO) wavelength (attributed to the Berreman mode) and the transverse optical (TO) wavelength (resulting from asymmetric Fabry–Pérot [FP] resonance). Despite their structural simplicity, the proposed emitters demonstrate versatile wavelength-, angle-, and polarization-selective optical functionalities across the long-wavelength infrared (LWIR) spectrum. We select a 1,000-nm SiO_2_/100-nm Al structure to construct the deep-subwavelength thermal emitter, which simultaneously generates 2 absorption peaks within the 7.5- to 14-μm range, corresponding to the working wavelength of the LWIR camera. With the thermal emitter serving as a physical-layer key, we build a high-security cryptographic communication scheme. The encrypted information is directly transmitted to the recipient, who can decrypt the message using a preset code and a physical-layer key. By integrating digital and physical layer encryption, the security of information transmission is markedly enhanced, establishing a blueprint for encryption systems and optically reconfigurable security frameworks in next-generation IR communication networks.

## Results and Discussion

**Fig. 1.**
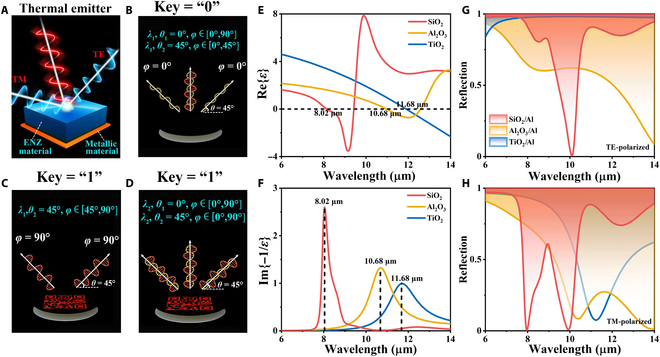
(A) Schematic of proposed bilayer thermal emitter with angle- and polarization-sensitive features, which consists of an epsilon-near-zero (ENZ) polar dielectric layer on a bottom metallic mirror. (B to D) The optical properties of the structure under various detection conditions and the corresponding code determined by the appearance (“1”) or the disappearance (“0”) of the pattern. (E and F) Permittivity of SiO_2_, Al_2_O_3_ [46], and TiO_2_ [46] in the long-wavelength infrared (LWIR) region. (G and H) Reflection spectra of transverse electric (TE) and transverse magnetic (TM) polarizations in the region of LWIR at Berreman thickness with corresponding incident angles of 45°, 55°, and 73°.

The proposed dual-wavelength narrowband thermal emitter consists of a metallic layer and an ENZ material layer (Fig. 1A). Figure 1B to D show its dual-peak thermal emission behaviors under various detection conditions. The first peak exhibits strong angular and polarization dependence, vanishing under normal incidence (0°) or transverse electric (TE) polarization but appearing at 45° incidence with transverse magnetic (TM) polarization. In contrast, the second peak remains stable, independent of angle or polarization. Under multilevel detection conditions (directionality *θ*, polarization *φ*, and wavelength *λ*), the emitter displays 2 distinct patterns, enabling a binary encoding mechanism (key = “1” for the presence and key = “0” for the absence of a pattern). For TM-polarized waves, a structure of a thin polar dielectric layer upon a metallic substrate exhibits LO and TO resonances. As demonstrated, the thin polar dielectric structure with an optimal Berreman thickness (*t*_B_) shows high absorption near the LO phonon-polariton resonance wavelength under TM-polarized oblique incidence. According to the Fresnel formula, *t*_B_ can be calculated as follows [42]:

$$t_{B}=\frac{\lambda }{2\pi }\frac{\text{cos}\theta }{{\text{sin}}^{2}\theta }{\left(\text{Im}\left\{\frac{-1}{\varepsilon }\right\}\right)}_{\max}^{-1}.$$ (1)

Here, *λ* represents the wavelength of the LO phonon resonance, *θ* is the incident angle (with *θ* = 0° denoting normal incidence), ε denotes the complex dielectric constant, and ${\left(\text{Im}\left\{-1/\varepsilon \right\}\right)}_{\max}$ represents the peak value of the energy loss function [42]. An asymmetric FP resonance near the TO wavelength generates the other absorption peak. This resonance arises from a dielectric layer stacked on a metallic reflector, differing fundamentally from conventional symmetric FP cavities [43,44]. Conventional symmetric FP resonators typically use macroscale or wavelength-scale cavities. In contrast, our design employs a deep-subwavelength cavity whose thickness is far below the diffraction limit. This subwavelength thickness leads to a negligible propagation phase shift, enabling the asymmetric FP structure to exhibit exceptional angular independence, maintaining near-constant absorption efficiency across wide incidence angles [45].

Accordingly, as for ENZ material, LO and TO resonances can be enhanced by adjusting the polar dielectric layer thickness. When the physical thickness for FP resonance coincides with the Berreman thickness (*t*_B_), absorption is simultaneously enhanced at certain angle near the LO wavelength (Berreman mode) and TO wavelength (FP resonance). To design an angle- and polarization-selective dual-wavelength emitter, we choose several ENZ materials with a *λ*_ENZ_ in the LWIR region, such as SiO_2_ (*λ*_ENZ_ at 8.02 μm), Al_2_O_3_ [46] (*λ*_ENZ_ at 10.96 μm), and TiO_2_ [46] (*λ*_ENZ_ at 11.9 μm). For the thin polar dielectric layer on a metallic substrate structure mentioned above, the absorption peak thickness induced by LO resonance is proportional to Im{−1/*ε*}, reaching its maximum at the wavelength where the real part of the permittivity approaches zero (*λ*_ENZ_), as exhibited in Fig. 1E and F. Reflection spectra for TE and TM polarization in Fig. 1G and H were obtained via transfer matrix method (TMM) simulations. The Berreman thicknesses for SiO_2_, Al_2_O_3_, and TiO_2_ were all set to 1,000 nm, with incident angles of 45°, 55°, and 73°, respectively. Here, SiO_2_ is capable of exciting Berreman modes (TM polarization) and FP resonance simultaneously within the LWIR region, while Al_2_O_3_ and TiO_2_ cannot meet the demand. This observation suggests that not all ENZ materials can simultaneously satisfy the 2 resonance conditions within this spectral range. Therefore, for practical observation by an LWIR camera, a 1,000-nm SiO_2_/100-nm Al bilayer structure (Fig. S1) that can generate 2 absorption peaks in the 8- to 14-μm range was thus chosen.

**Fig. 2.**
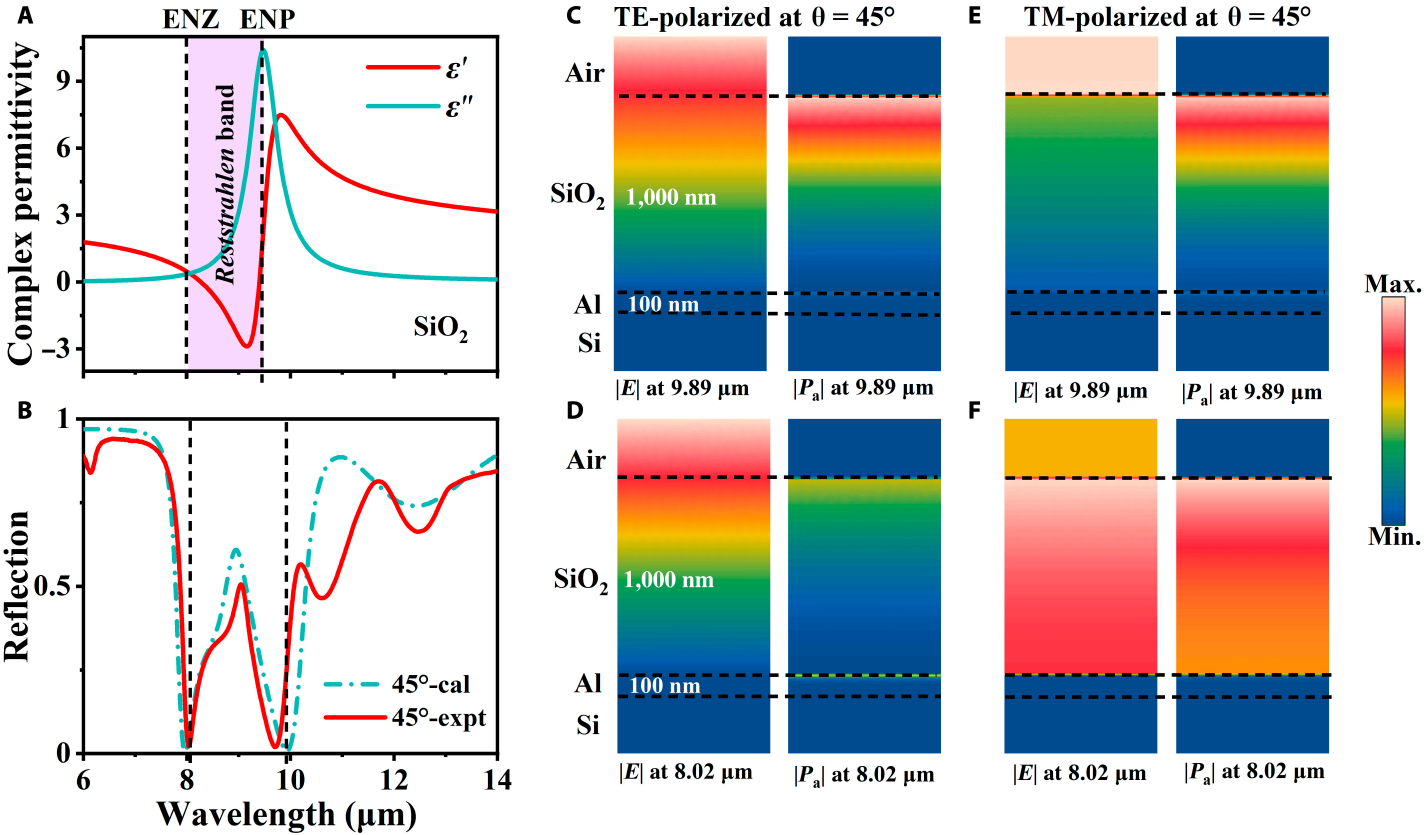
(A) Real and imaginary dielectric functions of SiO_2_ with 2 spectral positions of ENZ (at $\lambda_{\mathrm{ENZ}}=8.02\;\mu m$) and epsilon near pole (ENP) (at $\lambda_{\mathrm{ENP}}=9.89\;\mu m$). (B) Experimental and simulated reflectance of a sample with the wavelength for TM polarization at an incident angle of 45°. Here, the purple shaded regions denote the “*Reststrahlen* band”, which is bounded by ENZ and ENP wavelengths. (C to F) The normalized electric field amplitudes and the time-averaged power dissipation density are shown for the sample at an incident angle of 45° and wavelengths of 8.02 and 9.89 μm for (C and D) TE polarization and (E and F) TM polarization.

To properly explore the optical reflection behaviors of fabricated SiO_2_ films on an Al mirror, the optical parameters of the SiO_2_ thin films were firstly determined using IR spectroscopic ellipsometry. As depicted in Fig. 2A, the permittivity of SiO_2_, retrieved from variable-angle spectroscopic ellipsometry data (VASE; Fig. S2), which suggests 2 distinct features: ENZ (*λ*_ENZ_ = 8.02 μm) and epsilon near pole (ENP) (*λ*_ENP_ = 9.89 μm). Figure 2B shows the experimental and simulated reflectance of the thermal emitter at TM polarization and an incident angle of 45°. Evidently, the 2 peaks are near the ENZ and ENP wavelengths. To verify that these 2 absorption enhancement peaks arise from Berreman and FP resonance, the field distributions were investigated [47]. As can be seen from the distribution of the time-averaged power dissipation density in Fig. 2C to F, most of the incident light energy is concentrated and dissipated by the SiO_2_ layer. As exhibited in Fig. 2C and E, the distribution of the time-averaged power dissipation density of the sample is similar, indicating that the high absorption enhancement at 9.89 μm is caused by polarization-insensitive asymmetric FP resonance. The distribution of electric fields in Fig. 2C exhibits a close resemblance to that in Fig. 2D, as the fields in TE polarization are predominantly governed by the asymmetric FP resonance effect. The normalized electric field amplitudes and the time-averaged power loss density in Fig. 2F are all different from those in Fig. 2C to E, implying that the resonance at 8.02 μm for TM polarization is caused by the Berreman mode. The magnetic field distribution also resembles that of the electric field, displaying similar characteristics, as shown in Fig. S3. As can be clearly seen in Fig. S4, the simulated electric fields exhibit exponentially oscillating decay behaviors along the negative direction of the *z* axis, indicating an effective FP-like wave behavior.

**Fig. 3.**
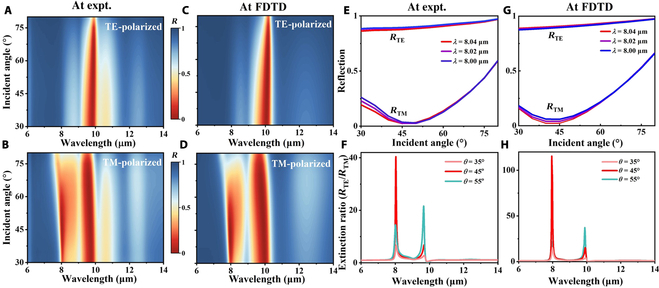
(A and B) Experimentally measured and (C and D) simulated reflection spectral mapping for TE and TM polarizations as a function of wavelength and incident angles (with 11 different incident angles *θ* from 30° to 80°). TE- and TM-polarized (E) experimental and (G) simulated reflectance for a 1,000-nm thick layer of amorphous SiO_2_ on top of a thick aluminum film. (F) Experimental and (H) simulated extinction ratios (*R*_TE_/*R*_TM_). FDTD, finite difference time domain.

Based on the above findings, the incident angle was varied from 30° to 80° in 5° increments to comprehensively investigate the reflection characteristics of the thermal emitter under both TE and TM polarizations. As illustrated in Fig. 3A to D, the reflectance at 8.02 μm for TE polarization remains near unity and insensitive to incident angle. In contrast, under TM polarization, the reflectance decreases dramatically with increasing incident angle, approaching near-zero reflection at larger angles. The corresponding reflectance spectra are presented in Fig. S5, corroborating these observations. Figure S6 displays the simulated and experimentally reflection spectra for TE and TM polarizations at *θ* = 10°. To evaluate the polarization selectivity of our thermal emitter, we introduce 2 figures of merit: the extinction ratio and efficiency. By definition, the extinction ratio is the ratio of output power to input power and efficiency is related to the input power [48]. Therefore, *R*_TE_ can be regarded as the efficiency and *R*_TE_/*R*_TM_ can be regarded as the extinction ratio. Figure 3E to H show the experimental and simulated reflectance data of this thermal emitter for TM-polarized and TE-polarized light (*R*_TM_ and *R*_TE_). The thermal emitter achieves an efficiency (*R*_TE_) of >90%, and the extinction ratio (*R*_TE_/*R*_TM_) becomes substantially large as *R*_TM_ nears zero. All results confirm that the thermal emitter achieves narrowband LWIR radiation with defined polarization angles and demonstrate its angle- and polarization-selective characteristics as viable features for IR encryption applications.

**Fig. 4.**
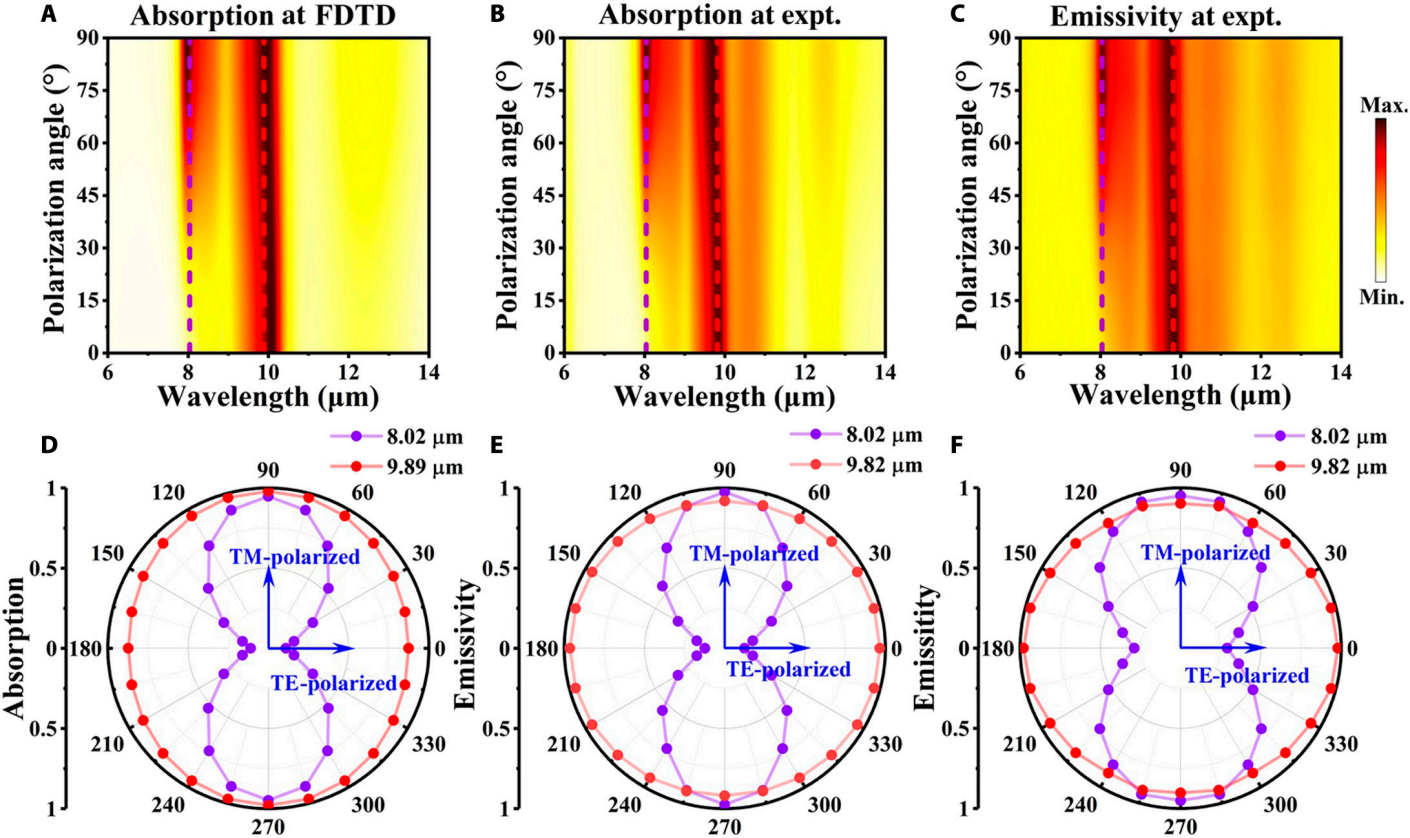
(A to C) The simulated absorption, experimentally measured absorption. and experimentally measured emissivity mapping spectra as a function of the wavelength and angle for unpolarized light at *θ* = 45°. (D to F) The corresponding polarization distributions at 8.02 μm (purple line) and 9.82(9.89) μm (red line) for the 3 cases discussed above.

In order to confirm the consistency between absorptivity and emissivity, absorptivity at 0° and 45° were calculated using the finite difference time domain method, while emissivity is obtained by normalizing the emission intensity of the thermal emitter measured by Fourier transform IR spectroscopy to that of a broadband blackbody. Figure 4A to C show the simulated/experimentally measured absorptivity and experimentally measured emissivity of the thermal emitter at 45° (corresponding data at 0° seen in Fig. S7). We performed Bland–Altman analysis to evaluate the agreement between experimentally measured absorption (*A*) and emission (*E*) rates at both 0° and 90° polarization angles (Fig. S8), and the plots demonstrate excellent consistency between absorption and emission measurements. The absorption spectra *A*(*λ*, *θ*, *φ*), which vary with wavelength and incident and polarization angles, were characterized using the following equation derived from Malus’s law [49,50]:

$$\begin{array}{c} A\left(\lambda ,\;\theta ,\;\varphi \right)=A\left(\lambda ,\;\theta ,\;\varphi =0{}^{\circ}\right){\text{cos}}^{2}\varphi +A\left(\lambda ,\;\theta ,\;\varphi =90{}^{\circ}\right){\text{sin}}^{2}\varphi , \end{array}$$ (2)

where *φ* represents polarization angles, *θ* denotes incident angles, *λ* is the wavelength of light, and $A\left(\lambda ,\;\theta ,\;\varphi =0{}^{\circ}\right)$ and $A\left(\lambda ,\;\theta ,\;\varphi =90{}^{\circ}\right)$ are absorption spectra for TE- and TM-polarized waves, respectively. The spectra demonstrate excellent agreement between absorptivity (*A*) and emissivity (*E*). This can be explained by Kirchhoff’s law, where absorptivity is equal to emissivity (*A* = *E*). Meanwhile, the emissivity of the thermal emitter was derived by calculating the reflection (*R*). Due to the presence of a thick Al layer (~100 nm) as a reflector, the transmittance can be considered negligible (*T* ≈ 0) [51]. Therefore, the reflectivity is given by *R* = 1 − *A* = 1 − *E*, meaning that the thermal emissivity could be also obtained from the corresponding reflectivity as shown in Fig. 3A and B. These results validate that designing a functional thermal emitter is equal to creating an absorber with similar properties. The absorptivity and emissivity of 9.82(9.89) μm in Fig. 4D to F almost remains unity at all polarizations and incident angles, while that of 8.02 μm increases as the polarization angle increases at *θ* = 45°. This indicates the polarization and direction selectivity of the thermal emitter, which are crucial for IR encryption–decryption applications.

**Fig. 5.**
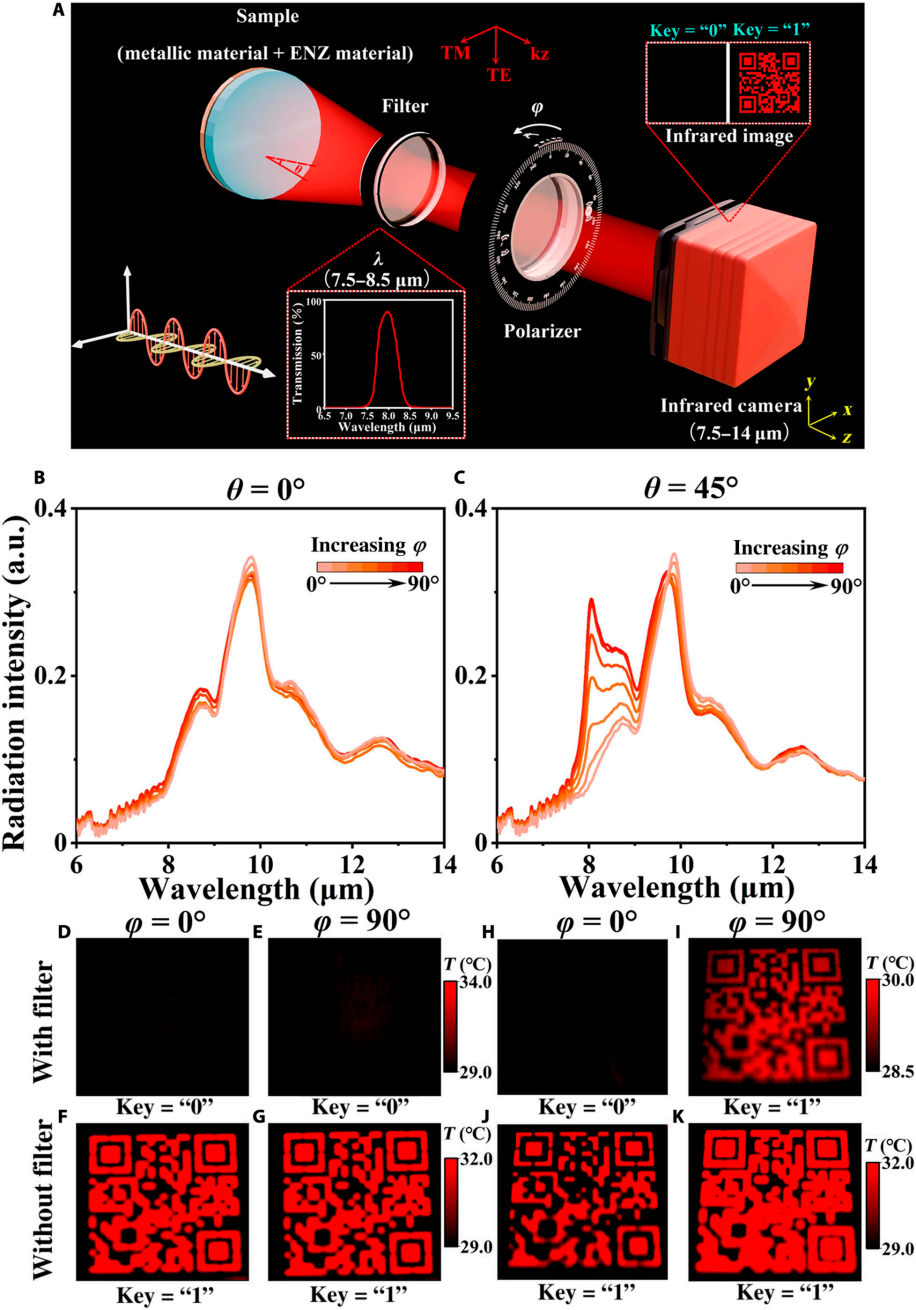
(A) Schematic of the angle- and polarization-selective infrared narrowband thermal emitters for infrared encryption–decryption applications. The thermal emitters sample emits dual-wavelength narrowband infrared light, which is then filtered by a 7.5- to 8.5-μm high-transmission filter and polarized by a polarizer. (B and C) The measured emission intensity spectra at *θ* = 0° and 45°. (D to K) Infrared images captured at *θ* = 0° or 45° and *φ* = 0° or 90°, with or without filter before the LWIR camera.

Utilizing the polarization and direction selectivity, Fig. 5A schematically illustrates a novel multichannel detection scheme. It consists of an LWIR camera (7.5 to 14 μm), an IR band-pass filter (wideband filters with a 500-nm bandwidth and an 8-μm central wavelength), and an IR polarizer (KRS-5 holographic wire grid polarizer). The IR image, whether containing Quick Response (QR) code information or not, can be acquired through precise control of 3 independent dimensions (directionality *θ*, polarization *φ*, and wavelength *λ*). The optical photograph of a fabricated large-area thermal emitter sample is also given, which has a diameter of 10 cm (4 inches) in Fig. S9. For demonstration purposes, we selected standard QR codes compatible with mainstream scanning applications (e.g., Alipay, WeChat, and mobile browsers) to ensure broad accessibility. The thermal radiation intensity data were obtained through Fourier transform IR spectroscopy measurements, as depicted in Fig. 5B and C, while the emissivity data are presented in Figs. S10 and S11. Subsequently, IR images were captured under different detection angles, with the results for *θ* = 0° and *θ* = 90° shown in Fig. 5D to K, and those for *θ* = 30° and *θ* = 60° provided in Fig. S12. The Stefan–Boltzmann law (shown as *P* = *EσT*^4^) can be used to understand the IR image result, which states that the radiated thermal energy (*P*) is directly related to the emissivity (*E*) and the surface temperature (*T*), where *σ* represents the Stefan–Boltzmann constant [52]. Since the emission intensity corresponds to the temperature data of IR images obtained by the LWIR camera, IR images can be encoded according to pattern visibility, where “0” represents no visible pattern and “1” exhibits a visible pattern, which endows it with the potential for encryption and decryption.

**Fig. 6.**
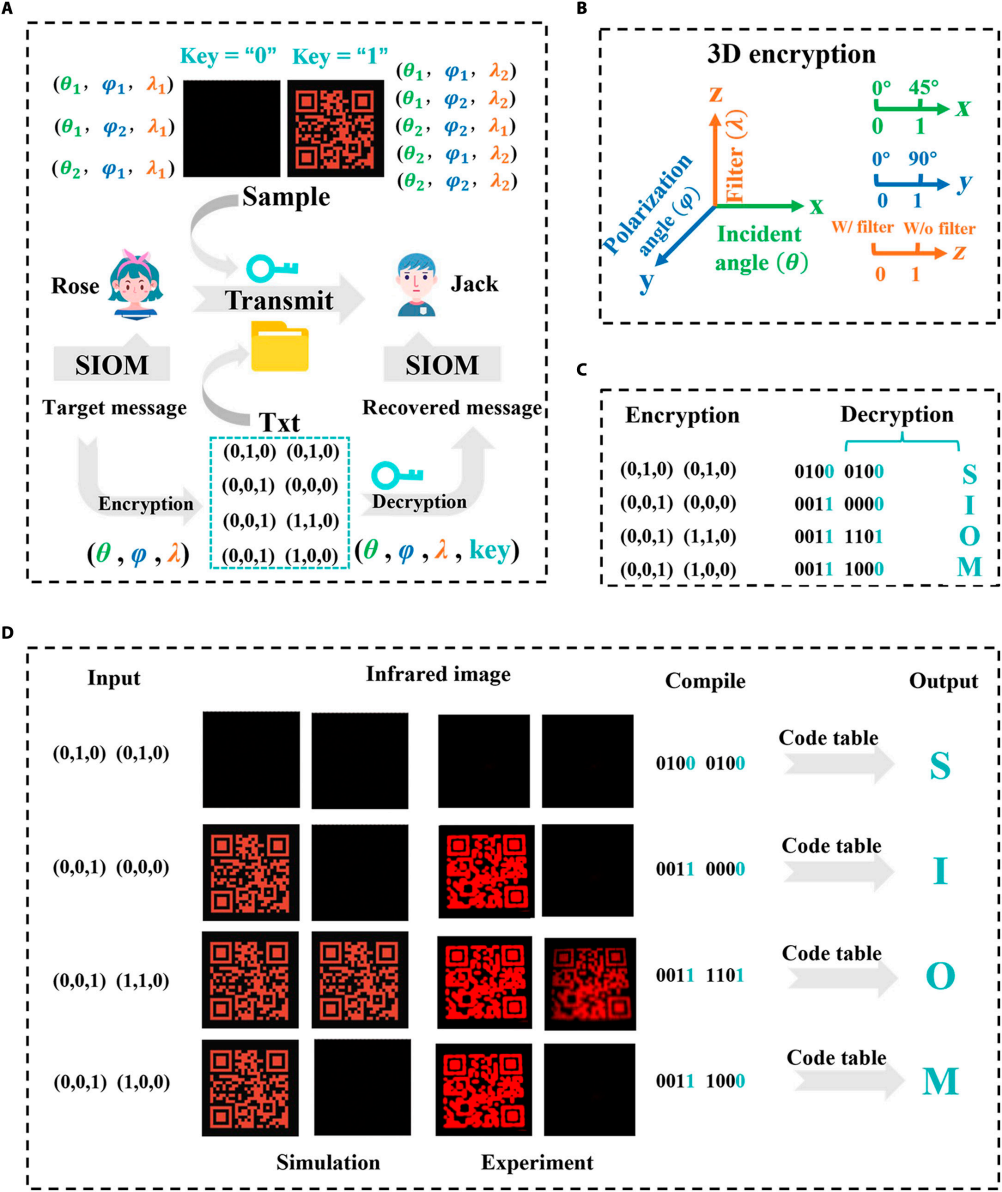
(A) The schematic depicts both the designed thermal emitter and its implementation as a physical-layer key for text message encryption. In the proposed encryption scheme, the target message and corresponding code table are encrypted into a text file, which can then be securely decrypted at the receiver end through authentication with the thermal emitter as the unique decryption key. (B) Schematic diagram of 3-dimensional (3D) encryption. (C) The programmatic process of information input (encryption) and output (decryption). (D) Quick Response (QR) code images under different input signals, showing the corresponding compilation, transcoding, and signal output processes. Decrypted information can be compressed or decompressed as required.

To improve the security of information transmission, we present a high-security cryptographic communication scheme based on the proposed thermal emitter. It can realize 8 independent conditions with different incident angle, polarization angle, and wavelength characteristics, as shown in the upper part of Fig. 6A. By using the thermal emitter as the physical-layer key, an encrypted communication scheme is established, as shown at the bottom of Fig. 6A. For example, if Rose wants to send Jack a message such as “SIOM”, this message can first be encrypted and converted into a txt file based on 8 independent channels and then sent to Jack via social networking software such as WeChat and email. Jack can recover the message with the thermal emitter physical key and the pre-agreed code table. Notably, the encryption process exhibits nonunique ciphertext generation for identical plaintext when different physical-layer keys are employed. This nondeterministic characteristic of the encrypted text files notably enhances information security by effectively resisting frequency analysis attacks.

This 3-dimensional multichannel encryption system (Fig. 6B) operates through the simultaneous modulation of 3 independent parameters, namely, incident angle (*θ* = 0° or 45°), polarization angle (*φ* = 0° or 90°), and wavelength (lower band *λ*_1_ or higher band *λ*_2_). A simplified schematic of the programmed process is drawn in Fig. 6C, while a detailed process is exhibited in Fig. 6D. For the input parameters (0,0,0), the corresponding detection condition requires setting the incidence angle to 0°, aligning the polarization angle at 0°, and operating the system with an optical filter, resulting in an IR image captured by the LWIR camera (Fig. 5D) from which the decoded key “0” is obtained. Similarly, after adjusting detection conditions according to the parameters (0,0,1), (0,1,0), (1,1,0), and (1,0,0), the corresponding IR images are acquired, enabling the extraction of their respective keys. The decoding process is achieved through the generation of 4-bit binary combinations from the input parameters in conjunction with the encryption keys. Two consecutive 4-bit combinations are subsequently concatenated to form an 8-bit binary sequence, which is uniquely mapped to corresponding alphabetical characters in the pre-defined code table, as illustrated in Fig. S13. As an exemplary demonstration of the decoding process, when the input sequence “010 010 001 000 001 110 001 100” is processed by our encryption system, the decrypted output “0 0 1 0 1 1 1 0” is obtained, mapping to the letters “S”, “I”, “O”, and “M”. This mechanism enables the transmission of encoded information, as the receiver deciphers the desired message by processing the sequence to obtain the corresponding key. While maintaining effectiveness, the current encryption approach shows susceptibility to frequency-based cryptanalysis. Future developments will incorporate advanced cryptographic protocols to strengthen system security. Notably, the encryption system is capable of generating nonunique ciphertexts from identical plaintexts through physical key modification, a characteristic that substantially enhances the security level of the cryptographic system and demonstrates revolutionize potential for propelling the advancement of next-generation encryption technologies.

## Conclusion

In summary, we report a novel type of bilayer narrowband thermal emitter, which is composed of a subwavelength SiO_2_ ENZ thin film on an optically thick Al mirror. Experimental results show that dual-band thermal emission is observed with angle and polarization selectivity in the LWIR spectrum. These emission responses are derived from the existence of multiple resonances including ENZ- and ENP-like modes combined with asymmetric FP modes. Numerical simulations of the electromagnetic field distributions in conjunction with TMM are performed to explain the optical properties; good agreement between the theoretical and experimental results is noted. Furthermore, from the application point of view, the proposed dual-band thermal emitter is suitable for IR encryption–decryption applications. Proof-of-concept results exhibit multilevel (directionality *θ*, polarization *φ*, and wavelength *λ*) thermal emission responses, which enable high-density IR encryption. This work suggests a simple yet robust lithography-free approach to fabricating dual-band thermal emitter and provides a blueprint for the development of IR multilevel encryption and anti-counterfeiting devices.

## Methods

### Numerical simulations

Full-wave numerical simulations were conducted using the TMM. The reflection and absorption spectra were calculated using TMM. The refractive indices of Al and SiO_2_ thin films were obtained from IR spectroscopic ellipsometry data analysis. Since the bottom Al film is much thicker than the penetration depth of light in the LWIR regime, the transmission (*T*) is nearly zero. Therefore, the absorption (*A*) can be calculated as *A* = 1 − *R*. The emissivity (*E*) of the sample was derived by calculating the absorption (*A*) according to Kirchhoff’s law, which states that the angular spectral absorptivity and emissivity must be equal (*E* = *A*). The electric field, magnetic field, and power loss density distributions were computed using the finite difference time domain method.

### Sample preparation

We fabricated a 4-inch photomask featuring a QR code corresponding to the official website of the Shanghai Institute of Optics and Fine Mechanics (SIOM). Following this fabrication process, a polished silicon wafer was first immersed in acetone and anhydrous ethanol successively, followed by ultrasonic treatment. After the cleaning procedure, the Al film was deposited at room temperature using electron beam evaporation (ZZS1100) at a deposition rate of 25 Å/s. Once the deposition was completed, the silicon wafer coated with 100 nm of aluminum was fetched out, and the photomask, which had a diameter of 10 cm (4 inches) (the QR code of SIOM’s official website), was carefully positioned over its surface. Finally, a 1,000-nm SiO_2_ thin film was deposited at room temperature at an evaporation rate of 5.8 Å/s.

### Material characterizations

The cross-sectional morphologies of the silicon oxide and Al films were characterized using a field-emission scanning electron microscope (Zeiss Auriga). Cross-sectional samples for scanning electron microscopy observation and energy-dispersive spectroscopy analysis were prepared using a focused ion beam system (Nova 600, Nanolab), enabling detailed structural and element analysis.

### Spectroscopic ellipsometry measurements

The optical characteristics of the aluminum and silicon oxide films were determined through the utilization of spectroscopic ellipsometry (IR-VASE MARK II, J.A. Woollam), covering a wavelength span from 1.7 to 30 μm. All of the fabricated aluminum and silicon oxide films were subjected to measurement by means of VASE at an incident angle of 55° (as depicted in Fig. S2). The refractive indices of aluminum and silicon oxide were extracted by fitting the VASE data with the Drude–Lorentz and Tauc–Lorentz oscillator models.

### Optical spectrum characterizations

The reflection spectra (*R*) and emissivity spectra (*E*) of the samples were analyzed and characterized with a Fourier transform IR spectrometer (Nicolet iS50, Thermo Fisher Scientific) with a variable-angle specular reflection accessory (VeeMAX III) for both TE- and TM-polarized light at incident angles from 30° to 80°. The thermal emission spectra were measured by positioning the emitter sample on a custom-designed hot plate maintained at 100, 150, and 200 °C, which served as an external radiation source for the spectrometer.

### IR images

Angle- and polarization-selective thermal IR images were acquired using a commercial LWIR camera (Fotric 228s, 7.5 to 14 μm) by manually adjusting the camera’s position and orientation while keeping the sample stationary. The setup included an external IR linear polarizer (Thorlabs WP50H-K), which was rotated to modulate the polarization state, and a wideband spectral filter (Thorlabs FB8000-500, central wavelength 8 μm, bandwidth 500 nm) to select the target wavelength range. The raw IR image data were processed using the AnalyzIR software package. The lower part of the obtained IR images may be a little blurred because of optical alignment and depth of field, but this does not affect the decrypted data integrity, as the key structural features remain resolvable.

## Data Availability

The data that support the findings of this study are available from the corresponding authors upon reasonable request.
