# Supplementary material for: An Angle- and Polarization-Selective Dual-Wavelength Narrowband Thermal Emitter for Infrared Multilevel Encryption
Source: Research (Wash D C). 2025 Jun 2;8:0719. doi: 10.34133/research.0719 (PMC12512774; doi:10.34133/research.0719)
Supplement: Supplementary 1 — Figs. S1 to S13 [file research.0719.f1.docx]

Supporting Information

**An angle- and polarization-selective dual-wavelength narrowband thermal emitter for infrared multilevel encryption**

Xuan Zhang, Zhengji Wen^*^, Qingzi Li, Zhanpeng Wang, Yusong Sheng, Zhengai Chen, Wenchao Zhao, Meng Guo, He Zhu, Ning Dai^*^, and Yuchuan Shao^*^

**Figure S1:**


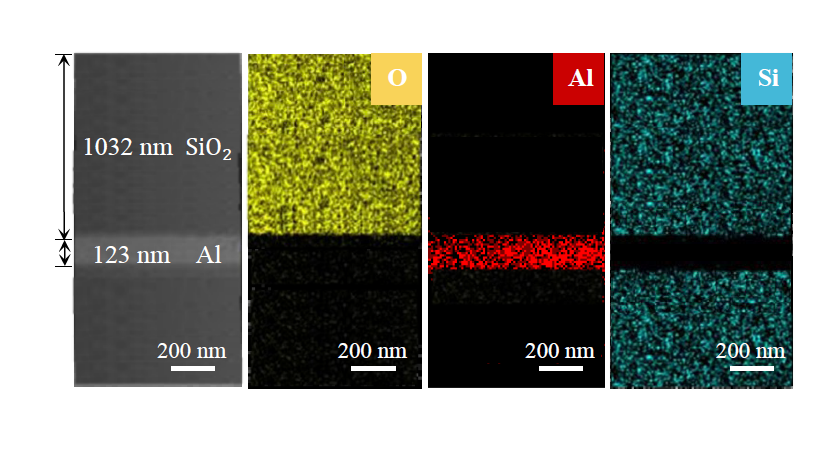


**Figure S1.** Cross-sectional SEM and EDS images of fabricated double-sided coating. The thickness of each layer is obtained from the SEM images. The yellow, red, and blue areas indicate O, Al, and Si, respectively.

**Figure S2:**


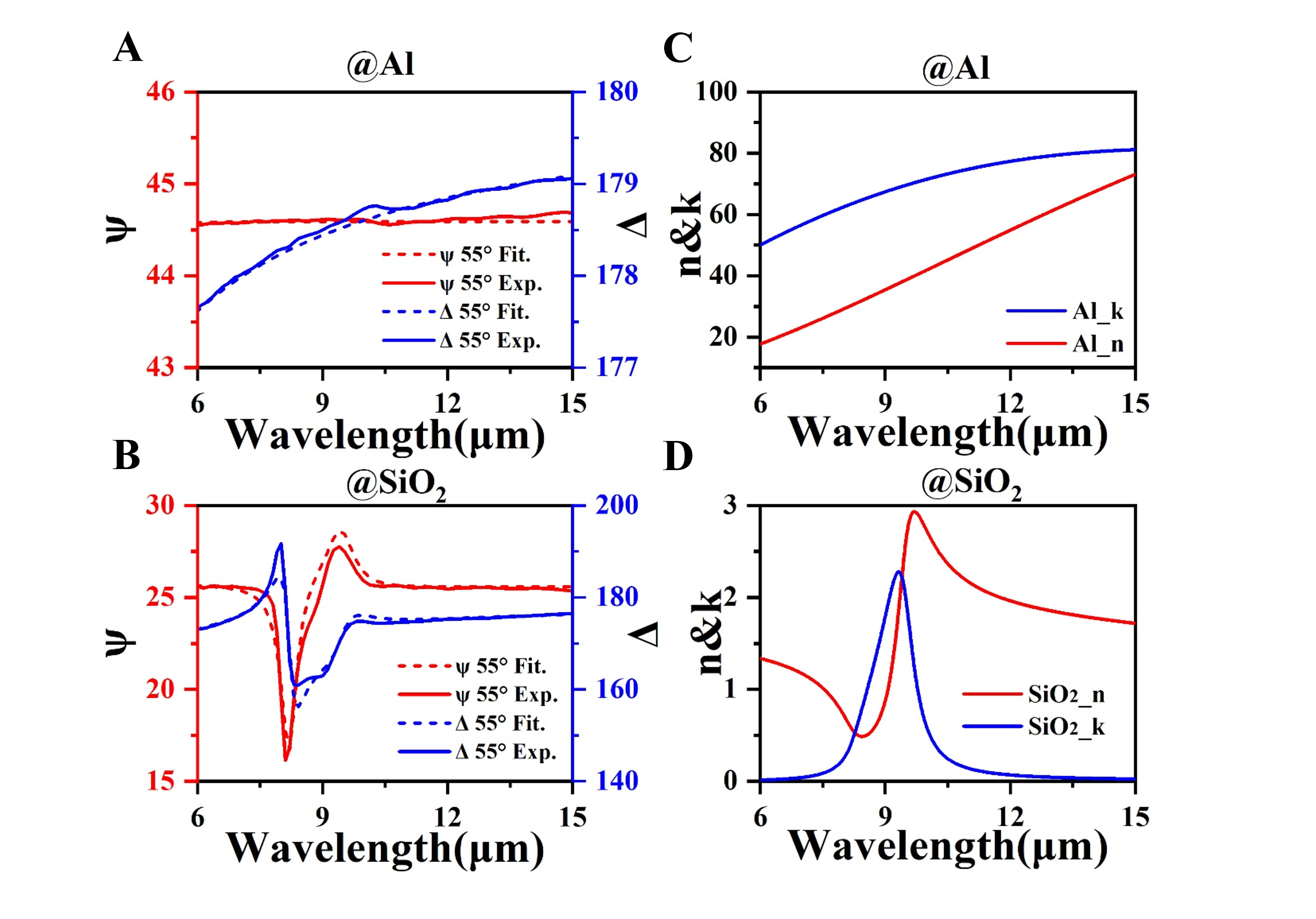


**Figure S2.** (A-B) Experimentally measured Ψ and Δ (solid curves) for SiO_2_ and Al spacers at angles of incidence *θ* = 55°, and the fitted results from the multiple Lorentz oscillators model (symbol curves). (C-D) Real and imaginary parts of refractive indices for the SiO_2_ and Al obtained from the ellipsometry data analysis.

**Figure S3:**


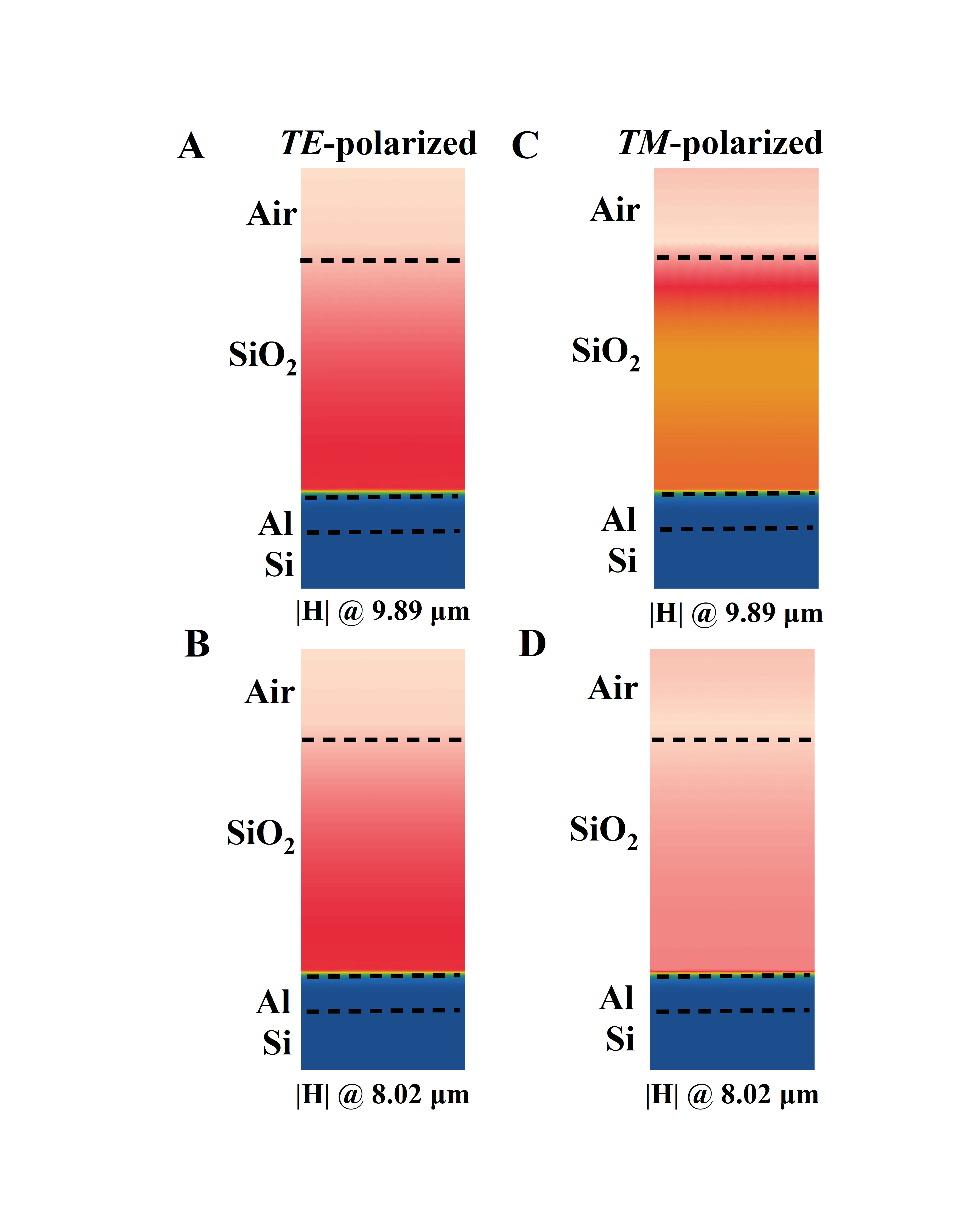


**Figure S3.** (A)-(D) The normalized magnetic field amplitudes are shown for the sample at an incident angle of 45° and the wavelength of 8.02 μm and 9.89 μm for (A)-(B) TE polarization and (C)-(D) TM polarization.

**Figure S4:**

**
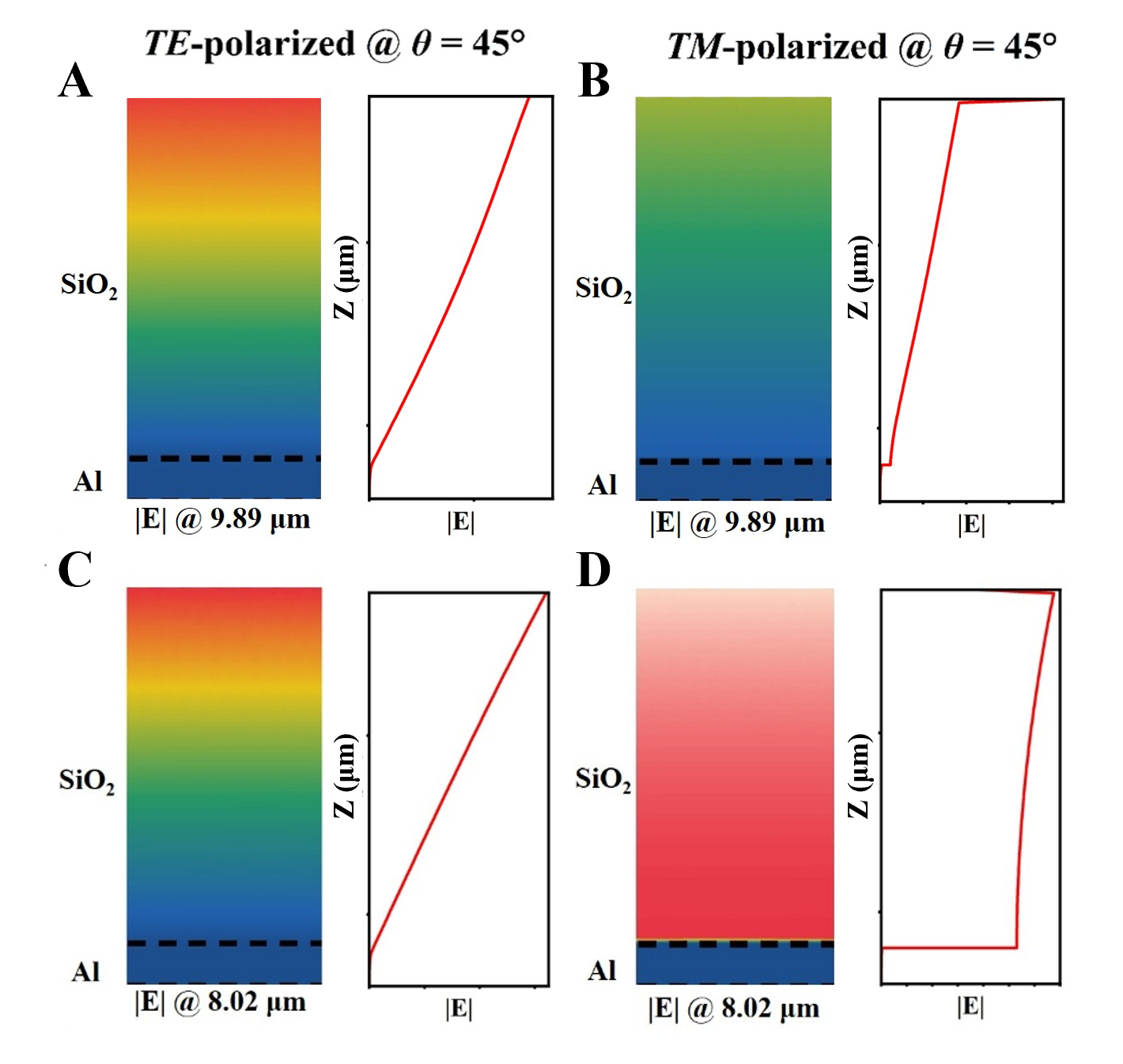
**

**Figure S4:** The 2D electric field for the sample at an incident angle of 45° and the wavelength of 8.02 μm and 9.89 μm for (A and C) TE polarization and (B and D) TM polarization. Red lines are the corresponding 1D cross-section plots.

**Figure S5:**


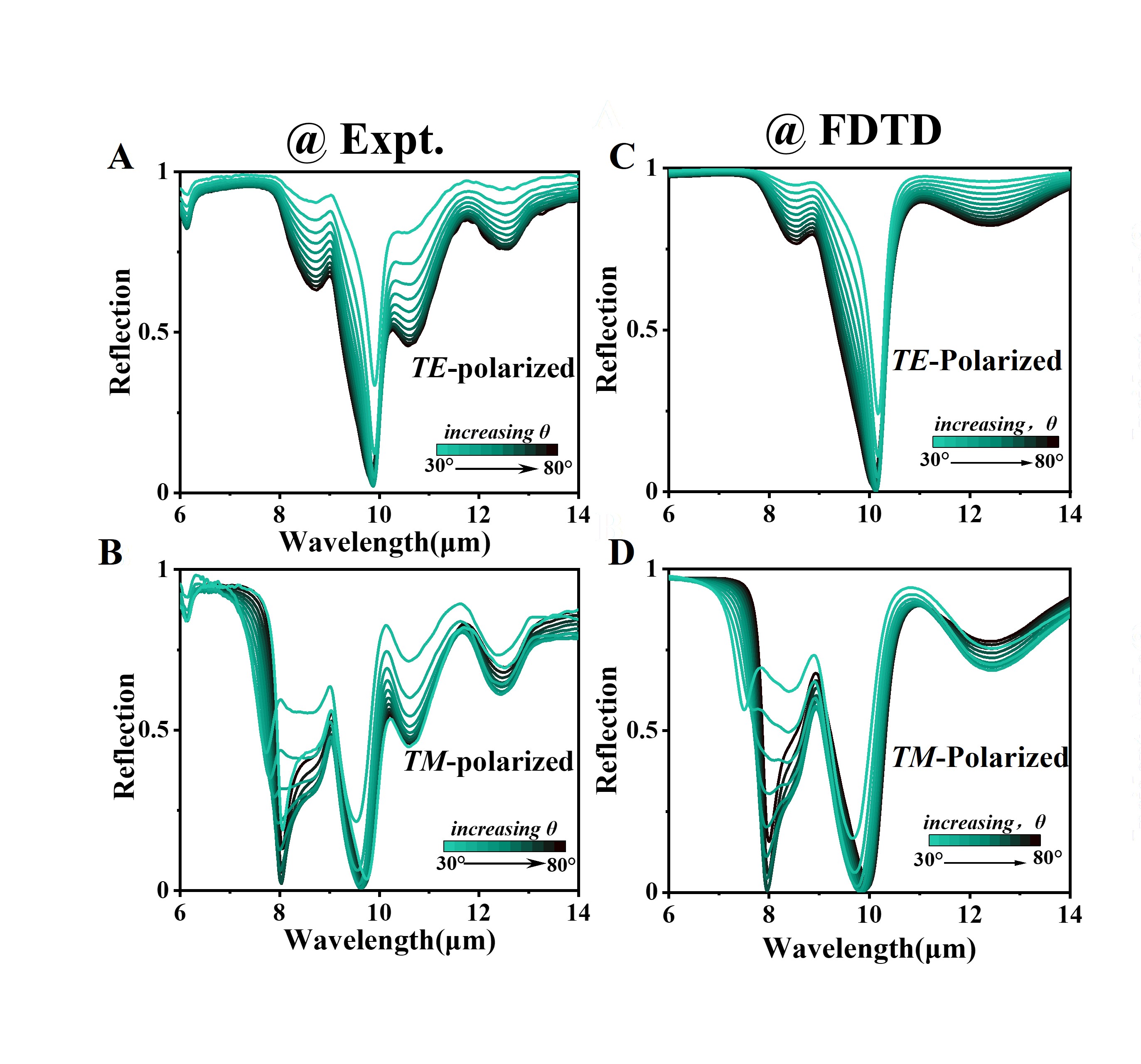


**Figure S5.** (A-B) Experimentally measured and (C)-(D) simulated reflection spectra for TE and TM polarizations as a function of wavelength and incident angles (with eleven different incident angles *θ* from 30° to 80°). The intensity of reflectance spectra at 8.02 μm decreases gradually as the incident angle increases from 30° to 80° for TM polarization. **Figure S6:**


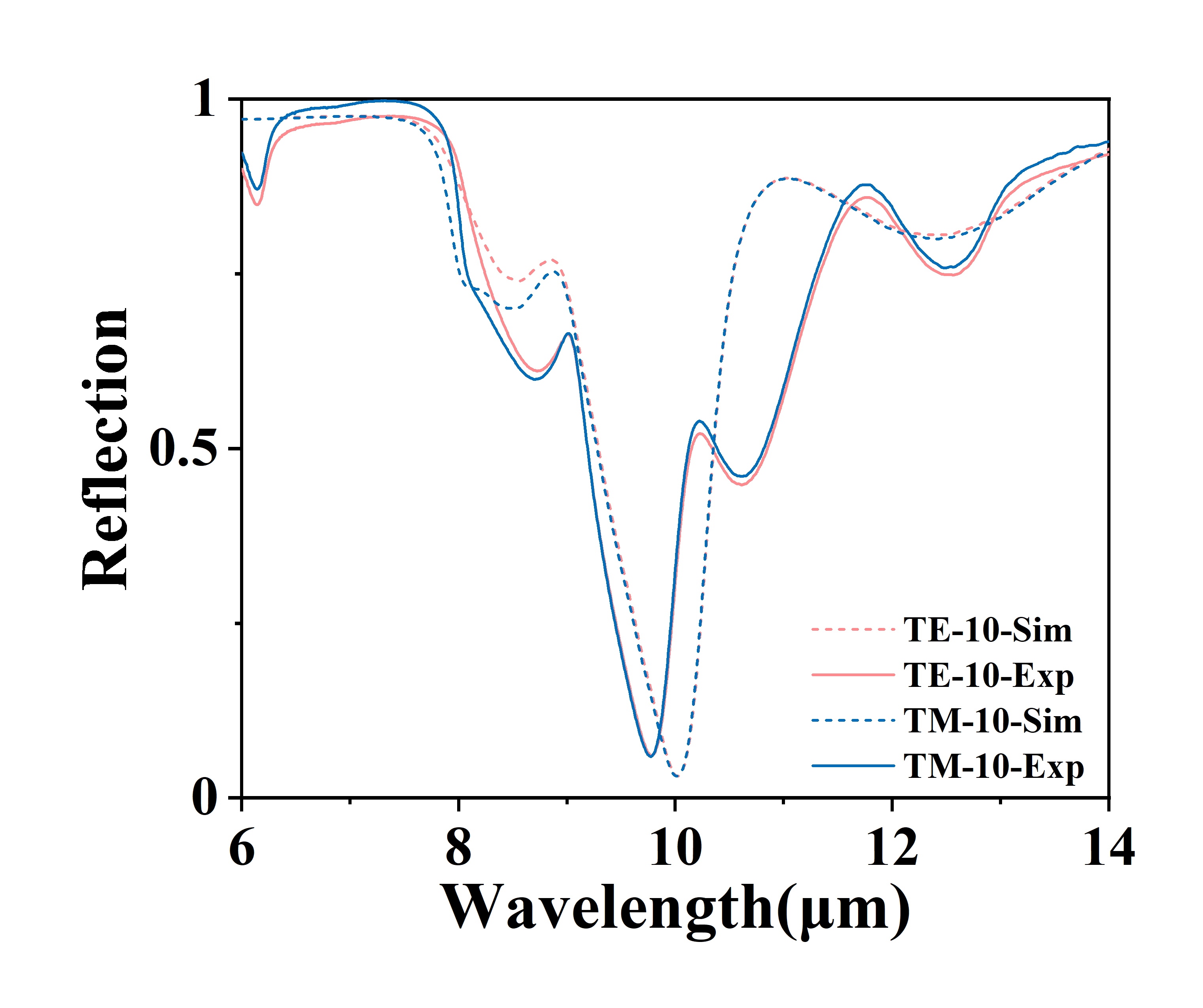


**Figure S6.** The simulated and experimentally reflection spectra for TE and TM polarizations at *θ* = 10°.

**Figure S7:**





**Figure S7.** (A)-(C) The simulated absorption and experimentally measured emissivity mapping spectra as a function of the wavelength and angle for unpolarized light at *θ* = 0°. Due to experimental constraints in measuring exact normal-incidence reflectivity, the experimentally measured absorption data were obtained at *θ* = 10°. (D)-(F) The corresponding polarization distributions at 8.02 μm (purple line) and 9.82(9.89) μm (red line) for the three cases discussed above.

**Figure S8:**


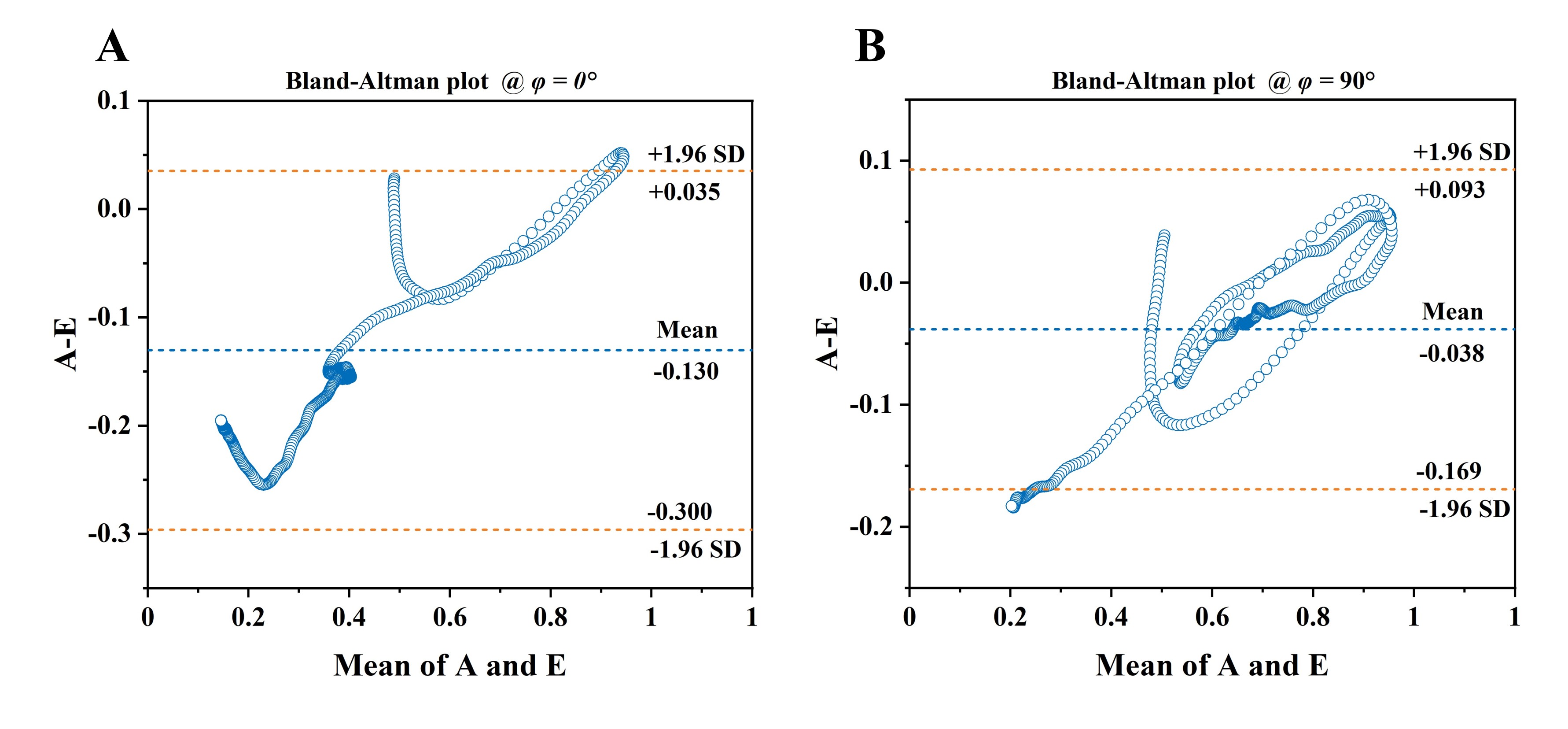


**Figure S8.** Bland-Altman plots comparing absorption (A) and emission (E) rates at (A) 0° and (B) 90° polarization angles. Blue horizontal lines indicate the mean difference between methods (A - E), while orange lines represent the 95% limits of agreement (mean ± 1.96 × SD of differences). The x-axis shows the average of paired measurements [(A+E)/2], and the y-axis displays the differences (A-E).

Difference：

$$D_{i}= A_{i}- E_{i}$$

Mean Bias：

$$Bias= \frac{1}{n}\sum D_{i}$$

Standard Deviation：

$$SD= \sqrt{\frac{1}{n-1}\sum\left( D_{i}-Bias \right)^{2}}$$

When the polarization angle was set to 0°, the observed bias was −0.13 with a standard deviation (SD) of 0.085. At a 90° polarization angle, the bias decreased to −0.038 (SD = 0.067). Given that the bias approaches zero, the SD remains relatively small, and the Bland-Altman plot shows that nearly all data points fall within the limits of agreement, we conclude that the experimental results are consistent with Kirchhoff’s law.

**Figure S9:**


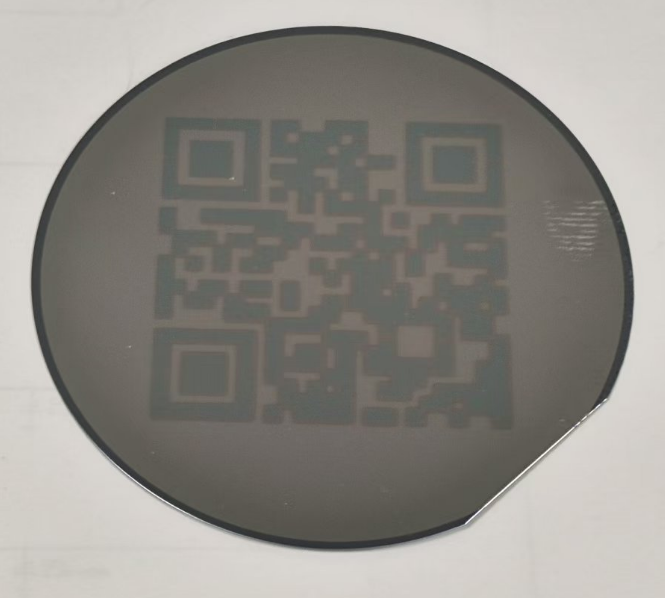


**Figure S****9.** Optical photograph of the fabricated large-area thermal emitter sample, which has a diameter of 10 cm (4 inches) (the QR code of the *Shanghai Institute of Optics and Fine Mechanics (SIOM)* official website).

**Figure S10:**


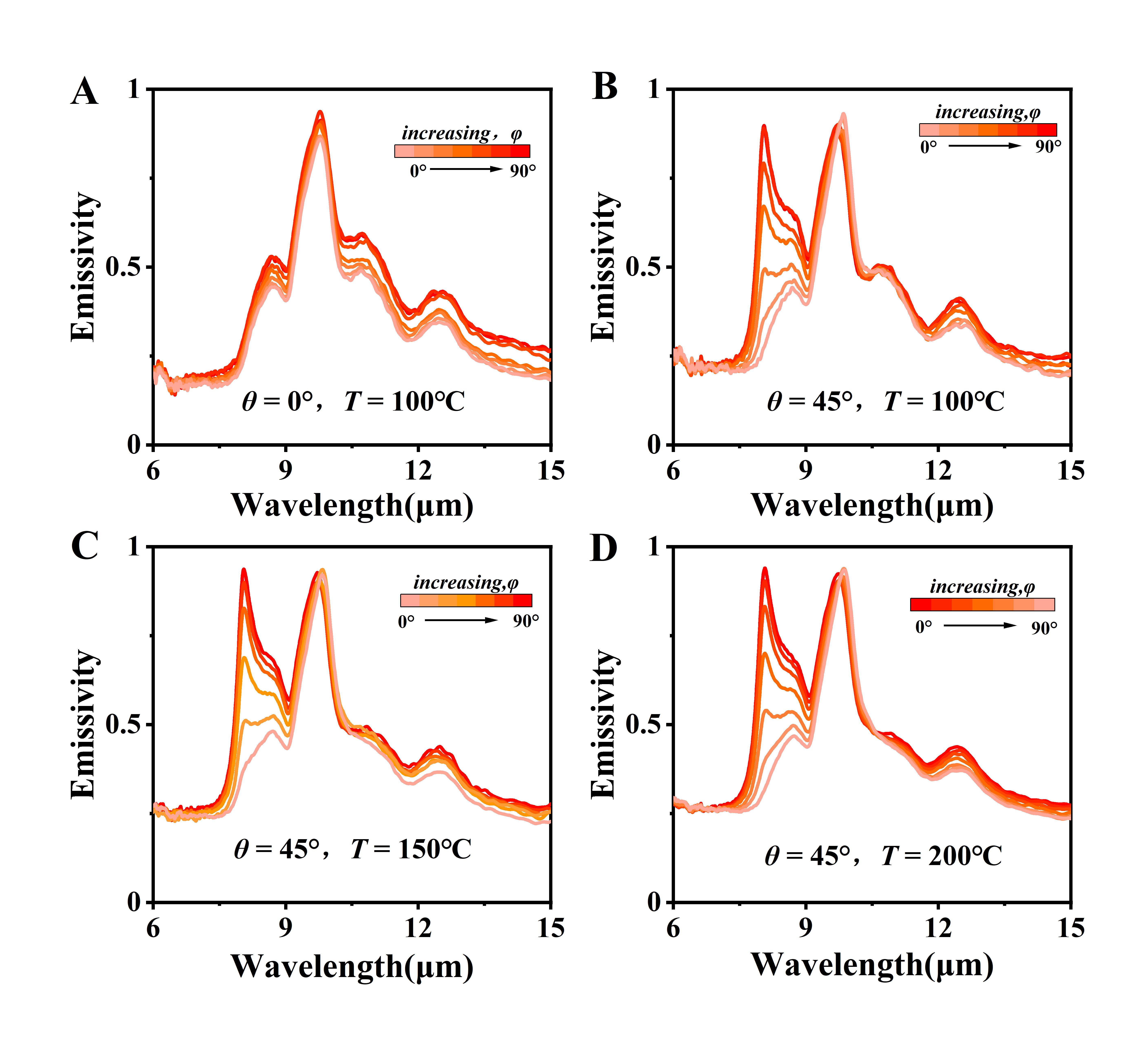


**Figure S10.** The measured emissivity spectra at *θ* = 0° and 45° when temperatures are 100 ℃, 150 ℃ and 200 ℃ with the polarization angle increases from 0° to 90° in steps of 15°.

**Figure S11:**


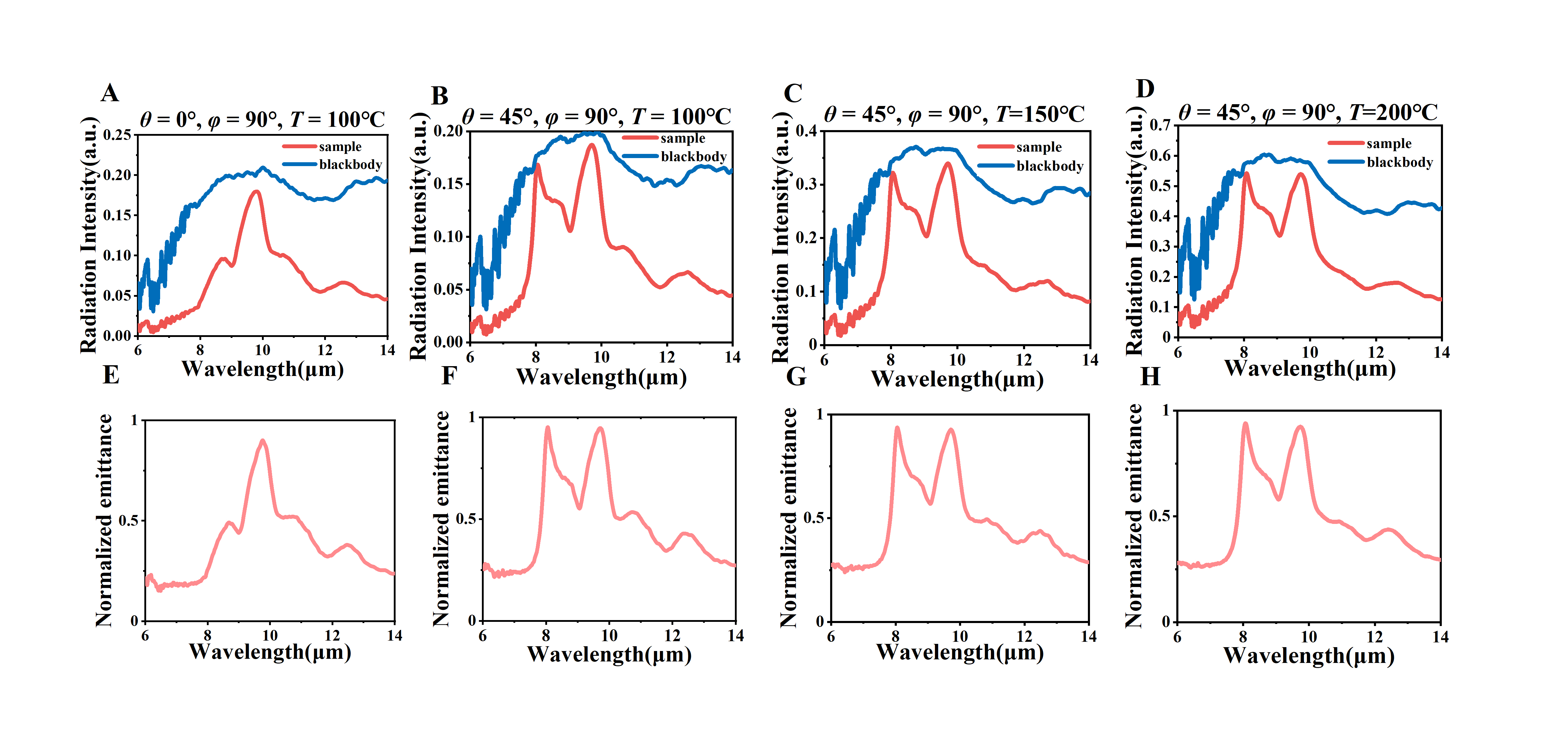


**Figure S11.** (A)-(D) thermal emission spectra of fabricated thermal emitter and a blackbody control sample at incident angles (*θ*) of 0° and 45° when temperatures are 100 ℃, 150 ℃ and 200 ℃ for TM polarization. (E)-(H) Normalized emittance of the fabricated thermal emitter.

**Figure S12:**


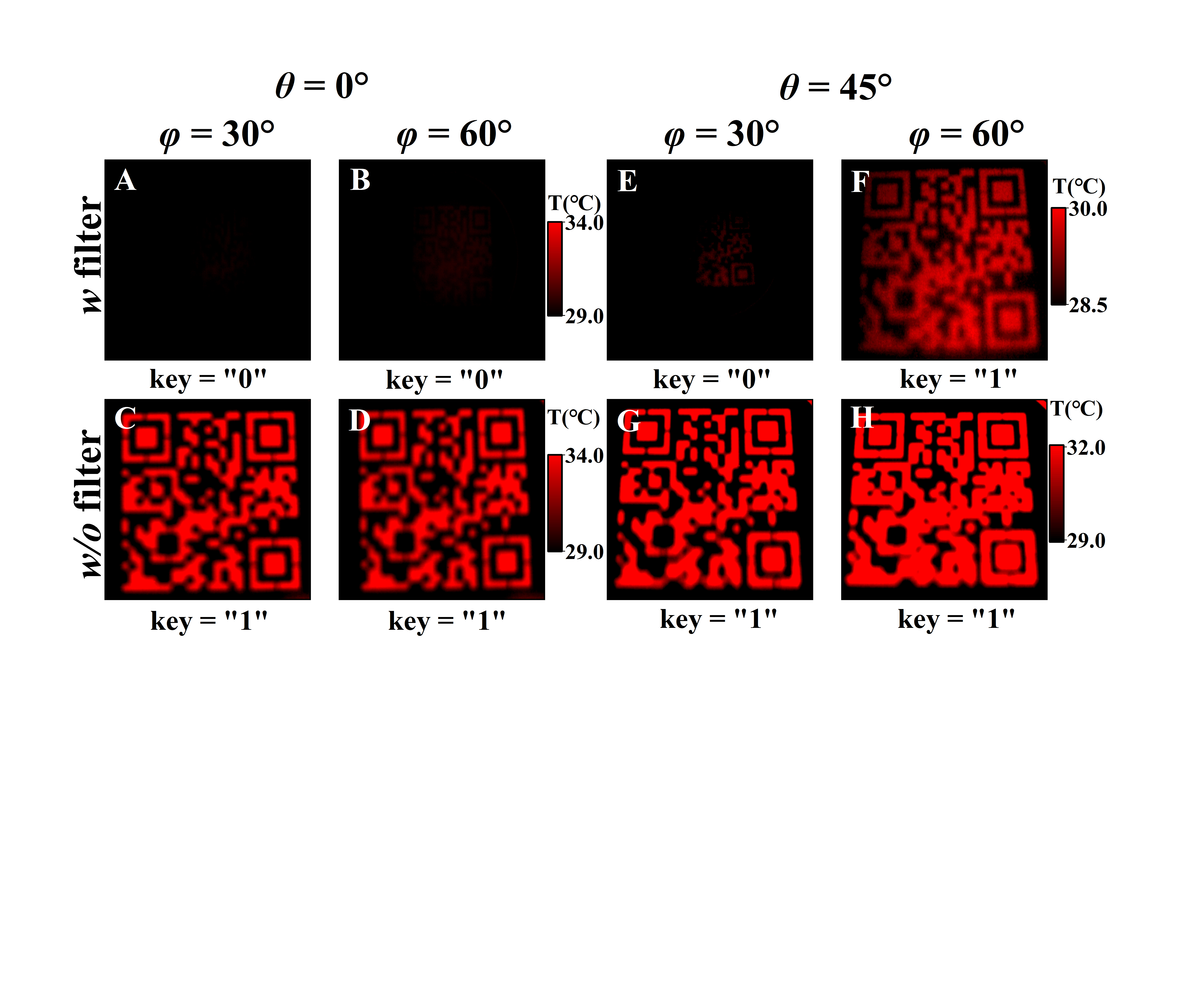


**Figure S12:** Thermal imaging characterization was performed on the fabricated wafer-scale patterned sample, which was designed with a QR code corresponding to the official website of the *Shanghai Institute of Optics and Fine Mechanics* (SIOM). The thermal measurements were conducted under varying experimental configurations, including incident angles (*θ*) of 0° and 45°, polarization angles (*φ*) of 30° and 60°, and with or without optical filter.

**Figure S13:**


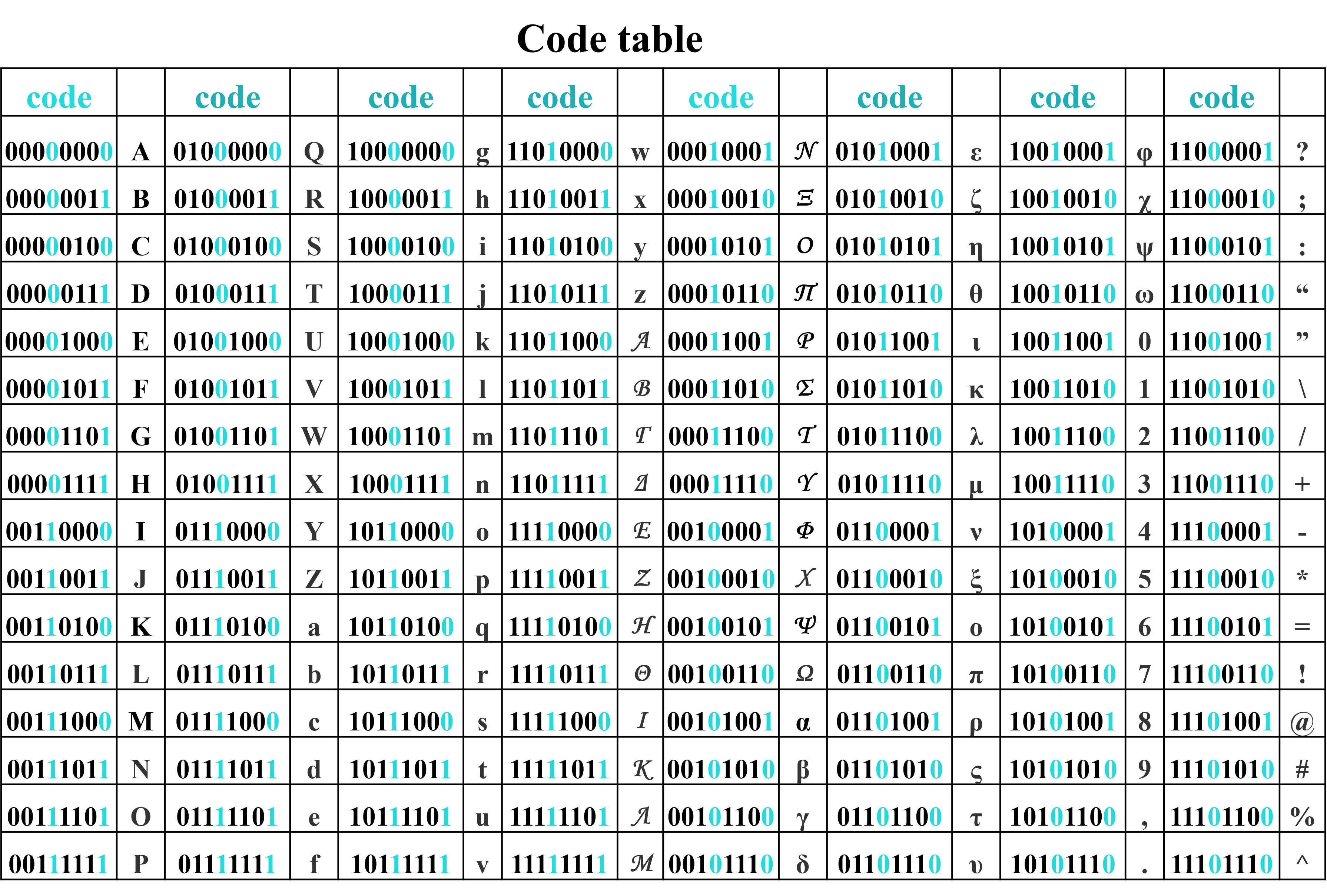


**Figure S13:** The code table of the high-security multi-channel cryptographic communication scheme.
